# Environmental microbiology going computational—Predictive ecology and unpredicted discoveries

**DOI:** 10.1111/1462-2920.16232

**Published:** 2022-10-11

**Authors:** Sara Hallin

**Affiliations:** ^1^ Swedish University of Agricultural Sciences Department of Forest Mycology and Plant Pathology Uppsala Sweden

## INCREASED DATA GENERATION AND DATA CRUNCHING

The fields of microbial ecology and environmental microbiology are producing loads of data, mainly nucleic acid sequence data due to the extensive use of amplicon sequencing and metagenomics, and an increasing use of transcriptomics. To increase our understanding of microorganisms in terrestrial ecosystems, multiple, concerted efforts to collect large numbers of samples for analyses of microbial communities were initiated already more than 15 years ago (Fierer & Jackson, 2006; Lozupone & Knight, 2007) but have really exploded the last years, with The Earth Microbiome Project Consortium being one of the first major endeavours for bacteria across all biomes (Thompson et al., 2017) and the work by Tedersoo et al. (2014) for soil fungi. The majority of the investigations have a biogeography focus based on a single sampling occasion and the word ‘global’ is frequently used in the titles of these soil microbial catalogues and surveys (Bahram et al., 2018; Delgado‐Baquerizo et al., 2018; Gobbi et al., 2022). Similar efforts have been done for many other biomes. Although largely descriptive, they have contributed to a better understanding of microbial diversity and the distribution of microbial taxa and their functions at an unprecedented spatial scale. Further, correlative analyses have indicted direct or indirect drivers of the observed patterns as well as the role of microbial communities for ecosystem functioning (Bahram et al., 2018; Delgado‐Baquerizo et al., 2020; Garland et al., 2021).

The massive amount of complex data is not only an opportunity but also a major challenge when it comes to meaningful interpretation. The field of computational biology, being the intersection of computer science and biology, is rapidly expanding and developing new methods for this purpose. Artificial intelligence (AI), including machine learning (ML) and to some extent also deep learning (DL) methods are promising for dealing with big data in microbial ecology and environmental microbiology (Ghannam & Techtmann, 2021; McElhinney et al., 2022). Especially ML approaches are increasingly adopted by ecologists and many of these methods will soon become routine tools for analyses of complex microbial omics data. They can be used to categorize and finds patterns in uncategorized data as well as analyse data that we know how to categorize. There are several advantages to using ML methods in microbiome studies, for example, they can deal with non‐linear relationships, make better use of the full depth of high‐dimensional data, and can be used to build predictive models based on environmental and community data.

Predictive modelling is very attractive in microbial ecology. Among the ML methods, random forests have become frequently applied in microbiome studies in the last decade (Jones et al., 2014; Ryo & Rillig, 2017). It is predominantly used for the identification of the best predictors for a given response variable and has for example been used to rank the environmental variables determining the major microbial phyla in wetlands (Bahram et al., 2022) and the diversity of ammonia oxidizing archaea across European soils (Saghaï et al., 2022), as well as the relative importance of biotic and abiotic controls of nitrous oxide emissions from agricultural soils (Jones et al., 2022). Random forest modelling can be very useful when studying remote areas that are difficult to sample, as exemplified by climate projections on microbial communities in the Antarctic Ocean (Tonelli et al., 2021). RF models can also show how predictions change over the range of each individual predictor variable, thereby giving the possibility to identify thresholds or tipping points (Apley & Zhu, 2020; Saghaï et al., 2022). Already in 2012, artificial neural networks were used to incorporate interactions among community members in models for predictions of microbial community composition in time and space based on environmental data (Larsen et al., 2012). A similar approach was used to predict the maize rhizosphere community at different plant development stages or growth conditions (García‐Jiménez et al., 2021). This type of approach can potentially assist in the microbiome engineering of important crops. However, with sequencing costs being relatively cheap, there is an increasing interest in using AI and microbiome data for microbiome‐based diagnostics as a means to address environmental challenges and advance management practices (McElhinney et al., 2022). Two recent examples of the latter are the use of soil microbiome data to predict the propensity for specific plant diseases in agriculture (Yuan et al., 2020) and soil health metrics (Wilhelm et al., 2022), which can be laborious and expensive to measure. Combining ML and microbiome data has further shown promising in environmental monitoring, tracing of contaminants and predictions of environmental quality (Sperlea et al., 2022; Techtmann & Hazen, 2016; Wheeler, 2019), which allows us to move away from indicator taxa or microbial biomarkers and instead use the full breath of information encompassed by the microbial community in a given site or sample.

## RE‐USING DATA AND SHIFTING TO A DATA‐DRIVEN COMPUTATIONAL SCIENCE

The large amounts of genetic data and corresponding meta‐data generated in microbiome studies are real treasures, especially when it comes to metagenomes and metatranscriptomes, and only a fraction of the information available has been explored. This data can be used for meta‐analyses to increase the scale of the study, but more importantly, it can be used to address other questions than those posed by the researchers that collected the original data. Making use of already published genome or sequence data in microbial ecology is not a new idea (Jones & Hallin, 2010) but now we have increasing possibilities to mine extremely large data sets (Coelho et al., 2022). Even more exciting are the possibilities to combine different types of data and information to go beyond the microbiome data. Integration of knowledge from diverse fields of research and the combination of microbiome data with other data from different sources have the potential to result in unexpected and unpredictable results, as well as new discoveries.

A recent example of re‐using and combining data is the work by Ke et al. (2022), who reanalyzed data in published datasets on the effects of pesticide application on soil microbial communities combined with information on the physical and chemical properties of the pesticides. By developing a ML model, they were able to show that physical pesticide properties largely explain the ecological impact of the pesticide. This information can guide the design of pesticide molecules to minimize environmental risk. In the field of precision agriculture, researchers have proposed the integration of AI and nanotechnology with disparate datasets to enable the design of nanoscale agrochemicals for sustainable food production (Zhang et al., 2021). In another study, geographic and meteorological data as well plant‐traits, land‐use type and microbial community data were used in a ML‐based prediction of grassland degradation, which is a multi‐factorial phenomenon not easily captured by a few variables (Yan et al., 2022). Combining datasets and using computational approaches can also be used to develop new diagnostic tools. For example, de Andrade et al. (2021) suggest the development of a soil quality index based on soil microbiome data, crop productivity and a range of abiotic environmental factors to improve crop production systems using AI. Data‐driven research relying on large, multiple, complex datasets and computational methods and capacity, as exemplified above, indicates a new paradigm in microbial ecology, and ecology in general (McCallen et al., 2019). We can anticipate new insights, similar to the leaps taken after advanced bioinformatics and multi‐omics approaches became an integral part of microbial ecology research.

Microbial ecology and environmental microbiology will follow the trajectory in life sciences and become increasingly computationally demanding, focusing on larger and also more complex sets of information. We are already seeing the laboratories being sparsely populated while students, postdocs, and researchers spend increasing amount of time in front of their computers organizing and analysing data. My crystal ball says that a shift towards a data‐driven rather than an experimental‐driven and data generating science, that depends on complex, big data, and advanced technologies, will be a game changer in microbial ecology and environmental microbiology. This development is already putting pressure on management, storage and sharing of data. Data‐driven microbial ecology research where different types of data are combined to consider the multidimensionality of ecosystems further suggests that students and researchers not only need to enhance their computational skills, but also skills in working interdisciplinary. Nevertheless, important discoveries should ideally be followed by experimental approaches to test hypothesis, determine causal relationships, and verify mechanisms. Already, experimental validation is definitely a bottleneck to close the circle in microbial ecology research and, although my crystal ball is a bit hazy here, it looks like this will become an even greater bottleneck in the era of big data and data‐drivenresearch in microbial ecology.

## Data Availability

There is no data associated with this article.
